# *Blastocystis* in the faeces of children from six distant countries: prevalence, quantity, subtypes and the relation to the gut bacteriome

**DOI:** 10.1186/s13071-021-04859-3

**Published:** 2021-08-12

**Authors:** Ondrej Cinek, Katerina Polackova, Rasha Odeh, Abeer Alassaf, Lenka Kramná, MaryAnn Ugochi Ibekwe, Edna Siima Majaliwa, Gunduz Ahmadov, Bashir Mukhtar Elwasila Elmahi, Hanan Mekki, Sami Oikarinen, Jan Lebl, Mohammed Ahmed Abdullah

**Affiliations:** 1 Department of Pediatrics, 2nd Faculty of Medicine, Charles University in Prague and University Hospital Motol, V Uvalu 84, Prague 5, Czech Republic; 2 Department of Pediatrics, School of Medicine, University of Jordan, Amman, Jordan; 3 Department of Pediatrics, Federal Teaching Hospital Abakaliki, Ebonyi State University, Abakaliki, Nigeria; 4 Muhimbili National Hospital, Dar-es-Salaam, Tanzania; 5 Endocrine Centre Baku, Str. I. Hashimov 4A, AZ1114 Baku, Azerbaijan; 6 Department of Paediatrics and Child Health, University of Khartoum, Faculty of Medicine, Khartoum, Sudan; 7 Sudan Childhood Diabetes Center, Khartoum, Sudan; 8 Faculty of Medicine and Health Technology, Tampere University, Tampere, Finland

**Keywords:** *Blastocystis*, Bacteriome, Type 1 diabetes, Asia, Africa

## Abstract

**Background:**

*Blastocystis* is a human gut symbiont of yet undefined clinical significance. In a set of faecal samples collected from asymptomatic children of six distant populations, we first assessed the community profiles of protist 18S rDNA and then characterized *Blastocystis* subtypes and tested *Blastocystis* association with the faecal bacteriome community.

**Methods:**

Stool samples were collected from 244 children and young persons (mean age 11.3 years, interquartile range 8.1–13.7) of six countries (Azerbaijan 51 subjects, Czechia 52, Jordan 40, Nigeria 27, Sudan 59 and Tanzania 15). The subjects showed no symptoms of infection. Amplicon profiling of the 18S rDNA was used for verification that *Blastocystis* was the most frequent protist, whereas specific real-time PCR showed its prevalence and quantity, and massive parallel amplicon sequencing defined the *Blastocystis* subtypes. The relation between *Blastocystis* and the stool bacteriome community was characterized using 16S rDNA profiling.

**Results:**

*Blastocystis* was detected by specific PCR in 36% (88/244) stool samples and was the most often observed faecal protist. Children from Czechia and Jordan had significantly lower prevalence than children from the remaining countries. The most frequent subtype was ST3 (49%, 40/81 sequenced samples), followed by ST1 (36%) and ST2 (25%). Co-infection with two different subtypes was noted in 12% samples. The faecal bacteriome had higher richness in *Blastocystis*-positive samples, and *Blastocystis* was associated with significantly different community composition regardless of the country (*p* < 0.001 in constrained redundancy analysis). Several taxa differed with *Blastocystis* positivity or quantity: two genera of Ruminococcaceae were more abundant, while *Bifidobacterium, Veillonella, Lactobacillus* and several other genera were undrerrepresented.

**Conclusions:**

Asymptomatic children frequently carry *Blastocystis*, and co-infection with multiple distinct subtypes is not exceptional. Prevalence and quantity of the organism clearly differ among populations. *Blastocystis* is linked to both faecal bacteriome diversity and its composition.

**Graphical Abstract:**

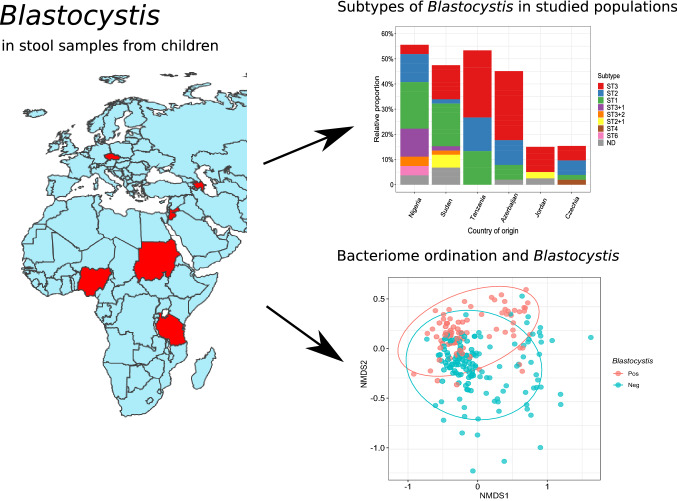

**Supplementary Information:**

The online version contains supplementary material available at 10.1186/s13071-021-04859-3.

## Background

The human gut microbiome consists of the microbial community (bacteria, archaea, viruses, fungi and protists), encompassing also their theatre of activity (microbial structures, metabolites, mobile genetic elements and relic DNA) [1]. The prime representative of the gut protozoa is *Blastocystis* sp., an anaerobic Stramenopile, a protist with a complicated life cycle and multifaceted morphology [2]. *Blastocystis* sp. is believed to be the most prevalent human gut eukaryote [3]. It is a symbiont: either a commensal [4] or a parasite whose clinical relevance is yet to be defined. The species is heterogeneous, comprising a growing number of subtypes (ST) with different biological properties encoded in their diverse genomes [5–7]. Many subtypes are known to colonize birds and mammals. Of the 22 subtypes defined as of 2018 [8], 10 were also found in humans, with ST3 or ST4 being most prevalent, followed by ST1 and ST2. The distribution of subtypes varies substantially among continents and countries—most prominently, the ST4 is found commonly in Europe but is rare elsewhere [9].

There are a growing number of studies on the prevalence, subtype distribution and also association of *Blastocystis* with the bacteriome composition. Moreover, *Blastocystis* has been studied in connection with two gastrointestinal primarily non-infectious diseases, inflammatory bowel disease (IBD, e.g. [4, 10]) and irritable bowel syndrome (IBS, systematic review of 17 published studies in [11]). Many questions, however, remain unresolved; the reported results widely differ based on age, selection of subjects and sampling strategy, and diagnostic and genotyping method.

We recently collected a multi-national set of samples from children and young persons without symptoms of gastrointestinal or other infection and characterized their bacteriome [12] and virome [13] to explore differences between children with recently diagnosed type 1 diabetes and matched controls. Here we revisited the sample set in order to define the faecal unicellular eukaryotic parasitome.

The aim of this work was to assess the prevalence, abundance and subtype representation of *Blastocystis* sp. among children and young persons from six different countries of three continents, and test the association between *Blastocystis* and stool bacteriome composition.

## Methods

**Fig. 1:**
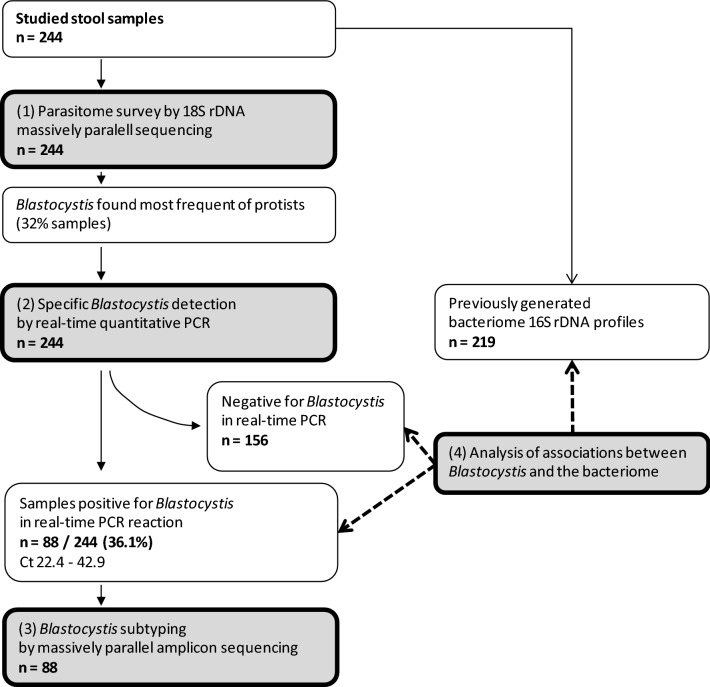
Workflow of the study

We tested 244 stool samples obtained from children and young persons from six countries. The molecular testing consisted of four steps. (i) First, the total parasitome was profiled by multiplex massively parallel amplicon sequencing of several regions of the 18S rDNA gene; this yielded a rough overview of unicellular parasites present in the samples. (ii) *Blastocystis* sp., having been identified as the by far most prevalent parasite, was tested and quantified using specific real-time PCR. (iii) *Blastocystis*-positive samples were classified into sequence types using massively parallel amplicon sequencing of an informative portion of the 18S rDNA. (iv) Finally, associations were explored between bacteriome 16S rDNA profiles and positivity or quantity of *Blastocystis*. The workflow is schematically shown in Fig. 1.

### Subjects and their samples

One stool sample was analysed per subject; the subjects were children and young persons from six countries participating in a case–control study on the microbiome in recently diagnosed type 1 diabetes and matched controls (children from Azerbaijan, Jordan, Nigeria, Sudan and Tanzania) [12] and in a study in Czech children with type 1 diabetes [14].

The demographic characteristics of the subjects are given in Table 1. Samples were taken using self-administered collection kits, immediately frozen in a home freezer and brought the next day to the physician's office. The cold chain was uninterrupted, with the exception of Sudanese samples that were briefly (< 24 h) exposed to refrigerator temperatures of 4–8 °C during air transport to the central laboratory because the use of dry ice at the Khartoum airport was not permitted.

**Table 1:** Characteristics of the studied subjects

	N of subjects	Females, *n* (%)	Age at sample, median, (IQR)	Of these, with type 1 diabetes, *n* (%)
Subjects from				
Azerbaijan	51	20 (39%)	10.2 (6.4–13.4)	18 (35%)
Czechia	52	17 (33%)	9.8 (8.1–12.7)	52 (100%)
Jordan	40	23 (58%)	10.8 (7.9–13.4)	20 (50%)
Nigeria	27	12 (44%)	14.5 (13.4–16.9)	13 (48%)
Sudan	59	34 (57%)	11.4 (7.4–12.9)	20 (34%)
Tanzania	15	9 (60%)	13.1 (11.1–14.6)	9 (60%)
Total	244	115 (47%)	11.3 (8.1–13.7)	132 (54%)

An aliquot of the stool sample (50–150 mg) was used for DNA extraction (Dneasy PowerSoil DNA isolation kit, MoBio/Qiagen) run on a Qiacube platform (Qiagen), whereas another aliquot was utilized for previously reported virome metagenomic sequencing [13].

### Molecular survey of protozoa using massively parallel profiling of the 18S rDNA gene

The molecular parasitome survey utilized five different reactions with amplification primers targeted to 18S rDNA (for sequences and PCR conditions kindly see Additional file 1: Supplementary Methods). Obtained PCR products were pooled together per sample, purified by Ampure XT (Beckman) in a 1:1 ratio and tailed using a limited counts of PCR cycles with Illumina combinatorial indices. After another cleanup and normalization using KAPA Library PCR quantification kit (Roche), the libraries were sequenced on a MiSeq instrument with the Reagent Kit v2, 2 × 250 bp (Illumina). The ensuing dataset was then processed using USEARCH61 pipeline, and 200 most prevalent operational taxonomic units (OTU) were manually classified by BLAST search to GenBank. Signals of the unicellular eukaryotes were expressed as the number of reads mapped to the particular organism, divided by the total reads per reaction.

### Detection and quantification of *Blastocystis* sp. using real-time quantitative PCR

Having identified *Blastocystis* sp. as by far the most frequent taxon among intestinal parasites, we tested and quantified it using real-time PCR with a specific hydrolysis probe. The real-time PCR was carried out with the primer-probe combination designed by Stensvold et al. [15] and a calibration curve from microscopically quantified xenic culture. The reactions were performed in duplicate. Because the PCR setup contained HotStar Taq polymerase chemistry (Qiagen, Hilden, Germany) instead of the originally used Platinum Taq polymerase (Invitrogen), the PCR programme started with 15 min at 95 °C to activate the polymerase and continued with 45 cycles of 15 s denaturation at 94 °C and 60 s of combined annealing and synthesis at 60 °C.

### Determining the Blastocystis sequence types using massively parallel amplicon sequencing

Samples yielding specific signal in *Blastocystis* real-time PCR were subsequently subtyped using massively parallel amplicon sequencing as specified by Maloney et al. [16], i.e. with primers originally developed by Santin et al. [17] extended with Illumina Nextera tails. Such a two-tier procedure using real-time PCR as the first step follows a previously published recommendation [15].

The amplicons generated by the tailed primers were purified and indexed by combinatorial indexing using the Nextera XT Index Kit v2 Set A and D (Illumina), which together enable multiplexing of 384 samples in a sequencing run. The indexed products were again purified, equalized and pooled. After supplementing with an addition of PhiX control to balance the amplicon signal, the libraries were sequenced on a MiSeq instrument with the Reagent Kit v2, 2 × 250 bp (Illumina). The sequencing yielded a mean of 4195 (interquartile range, IQR, 2793-5770) paired reads per sample. Of these, 95% (87–99%; mean, IQR) were *Blastocystis* amplicons. The modal sequence length of the *Blastocystis* amplicon after primer trimming was 449 bases (range 444–454), depending on the ST.

Sequencing files were downloaded from the Illumina BaseSpace cloud demultiplexed and trimmed of the sequencing adaptors. The sequencing reads were then processed using the 64-bit version of USEARCH10 suite [18]. The reads contained the amplification primers; because mismatches under primers have been documented in numerous sequence types, primers were trimmed. Reads were filtered with an expected error parameter *maxee* = 1.0. Unique sequences were then defined as zero-radius operational taxonomic units (ZOTU) [18], denoised and their counts entered into a cross-tabulation (ZOTU table). The taxonomy (to the level of genus) was defined using the Silva 18S eukaryotic database, and a few ZOTUs were removed that belonged to off-target amplicons, mostly of bacterial origin. The bioinformatic pipeline was run on an Amazon Elastic Cloud machine. The pipeline performed essentially identical steps as the one by Maloney et al. [16], with the exception of added primer trimming.

The above procedure identified 84 unique *Blastocystis* ZOTUs. Their sequence types were defined by clustering with a reference set of relevant representatives of selection of human subtypes, mostly submitted by Maloney et al. [16] (GenBank accessions MK244898–MK244953). The sequences were aligned in the Geneious programme version 8.1.9 (Biomatters Inc., New Zealand) using its proprietary algorithm and the ClustalW programme, distance matrix computed and phylogenetic tree drawn with HKY85 model, neighbor-joining algorithm, 1000 bootstrap resamplings and an outgroup of *Proteromonas lacertae* sequence (GenBank accession U37108.1, a distantly related gut flagellate of amphibians).

The ZOTU frequency table, ZOTU sequences, phylogenetic tree and sample metadata were then combined into a *phyloseq (SummarizedExperiment)* object [19] and the downstream analyses and visualizations were performed in R [20].

After the dataset was analysed on the level of ZOTUs, the analysis continued by reducing the excess intra-organismal variation using the *tip_glom* method of *phyloseq* with method set to agglomerative hierarchichal clustering by UPGMA (average linkage) and the height of cutting set to 0.03, a level corresponding to the similarity threshold used in other studies of *Blastocystis* (e.g. [21]). This may partly eliminate sequencing artefacts, although some of the intra-organismal variability between 18S rDNA gene copies is inevitably lost. To mitigate the unwanted effects of artefacts arising from index hopping (cross-bleed of signal between unrelated index combinations) on the measures of inter- or intra-species variation (i.e. on the count of OTUs in one sample), we imposed a 0.5% threshold for sequence type positivity. This is at least one order of magnitude above the level of artefactual signal from index hopping that we previously observed [22].

### Statistical analysis

Logistic regression model was used for comparing odds of being *Blastocystis* positive (any level of positivity, i.e. the outcome was defined as positive quantitative real-time PCR), whereas the quantities were modelled using linear regression with the decimal logarithm of estimated quantity as an outcome. The predictors tested in both models were nationality, gender and status of the subject (healthy or type 1 diabetes). Quantities of positive samples were compared by Kruskal-Wallis test and pairwise Wilcoxon test with Bonferroni correction for the number of comparisons.

### Analysis of associations between the bacteriome and Blastocystis positivity

Bacteriome profiles from 219 of the 244 samples were available from our previous studies [12, 14]. The raw bacteriome sequencing data from the two substudies were pooled and reanalysed using the DADA2 pipeline [23], and transferred into a *phyloseq* object [19].

Alpha diversity of the bacteriome (i.e. diversity within samples) was characterized using six different measures, and three models were used for assessment of the association with *Blastocystis*: (i) conditional logistic regression model with *Blastocystis* PCR positivity as a dichotomous outcome, with the diversity index as a predictor and nationality as a conditioning variable; (ii) linear model with the diversity index as outcome and *Blastocystis* positivity with nationality as predictors and (iii) the latter with logarithm of *Blastocystis* quantity as predictor.

Beta diversity (dissimilarity between two community samples) was assessed using the quantitative Bray-Curtis index. Ordination was performed using non-metric multidimensional scaling (NMDS) and samples were plotted by country of origin and *Blastocystis* positivity. The visually apparent separation of the bacteriome profiles by *Blastocystis* positivity or quantity was then tested by Permutational Multivariate Analysis of Variance (PERMANOVA) implemented in the *vegan* package as the *adonis* function [24], with 1000 permutations. Nationality, *Blastocystis* and the interaction term were group variables. Lack of difference of variances among groups of *Blastocystis* positivity and nationality was verified by the function *betadisper*. Further model was run with permutations restricted within the nationalities.

The differences in community composition were assessed by redundancy analysis (RDA) on Hellinger-transformed bacteriome abundance tables using *Blastocystis* positivity and nationality as predictors. Association of *Blastocystis* with abundance of individual bacterial taxa was then assessed using the *DESeq2* tool [25] embedded through the *phyloseq-to-deseq* function within the *phyloseq* package. Models were adjusted for nationality. *P* values were corrected for multiple testing using the Benjamini-Hochberg method implemented within the *DESeq2* package, and *p* < 0.05 was considered significant. Results were further filtered by abundance of the taxon (base abundance in the model > 1%) and for effect size (only taxa differing > twofold in either direction were retained).

## Results

### *Blastocystis *was the most often observed protist in stool samples

In the molecular survey of protozoa by 18S rDNA amplicon sequencing, the most often observed parasite was *Blastocystis*: its signal was noted in 77/244 (31.6%) samples. The survey further identified *Entamoeba* sp. (16/244, 6.6%), *Dientamoeba* sp. (13/244, 5.3%) and *Endolimax* sp. (8/244, 3.3%). The frequencies of detected unicellular parasites are broken down by nationality in Additional file 1: Table S1. Further signals observed in the 18S rDNA sequences belonged to human genome, food, bacteria, fungi and multicellular parasites, presumably from ova. The distribution of signal from gut parasites is schematically shown in Additional file 1: Figure S1.

### Real-time PCR detection and quantification of *Blastocystis* sp.

**Fig. 2:**
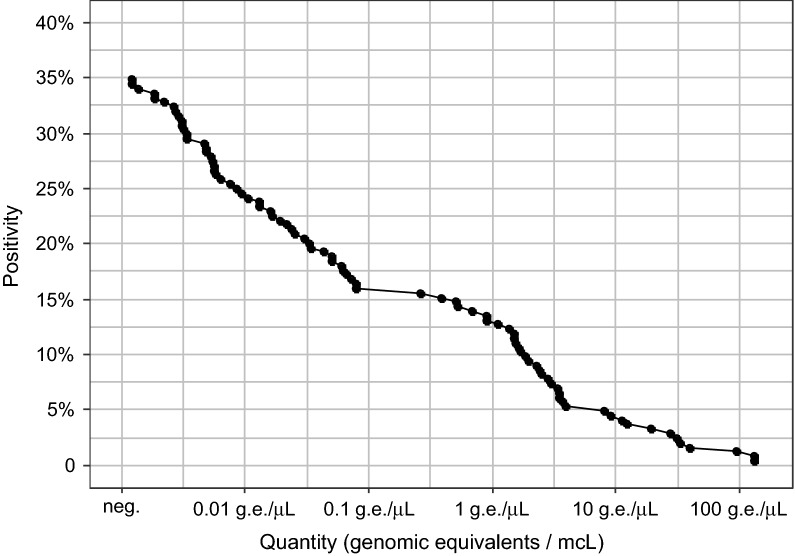
Distribution of quantity in *Blastocystis*-positive samples. The value shown is the proportion of samples exceeding the indicated threshold of positivity. The proportion of positive samples is plotted against the positivity threshold in a modified empirical cumulative distribution function graph

Specific real-time PCR reaction detected *Blastocystis* in 88/244 stool samples (36.1%). The *Blastocystis* quantities widely differed, the threshold cycle (Ct) of detection being from 42.8 to 22.4, which corresponds to quantities of < 1/100 of a genome/µl DNA to over 100 copies of *Blastocystis* genome/µl DNA (shown in the empirical cumulative distribution graph in Fig. 2). Of note, the ribosomal DNA is present at least in higher tens in each copy of the *Blastocystis* genome, so values well under one complete genome copy are plausible.

**Fig. 3:**
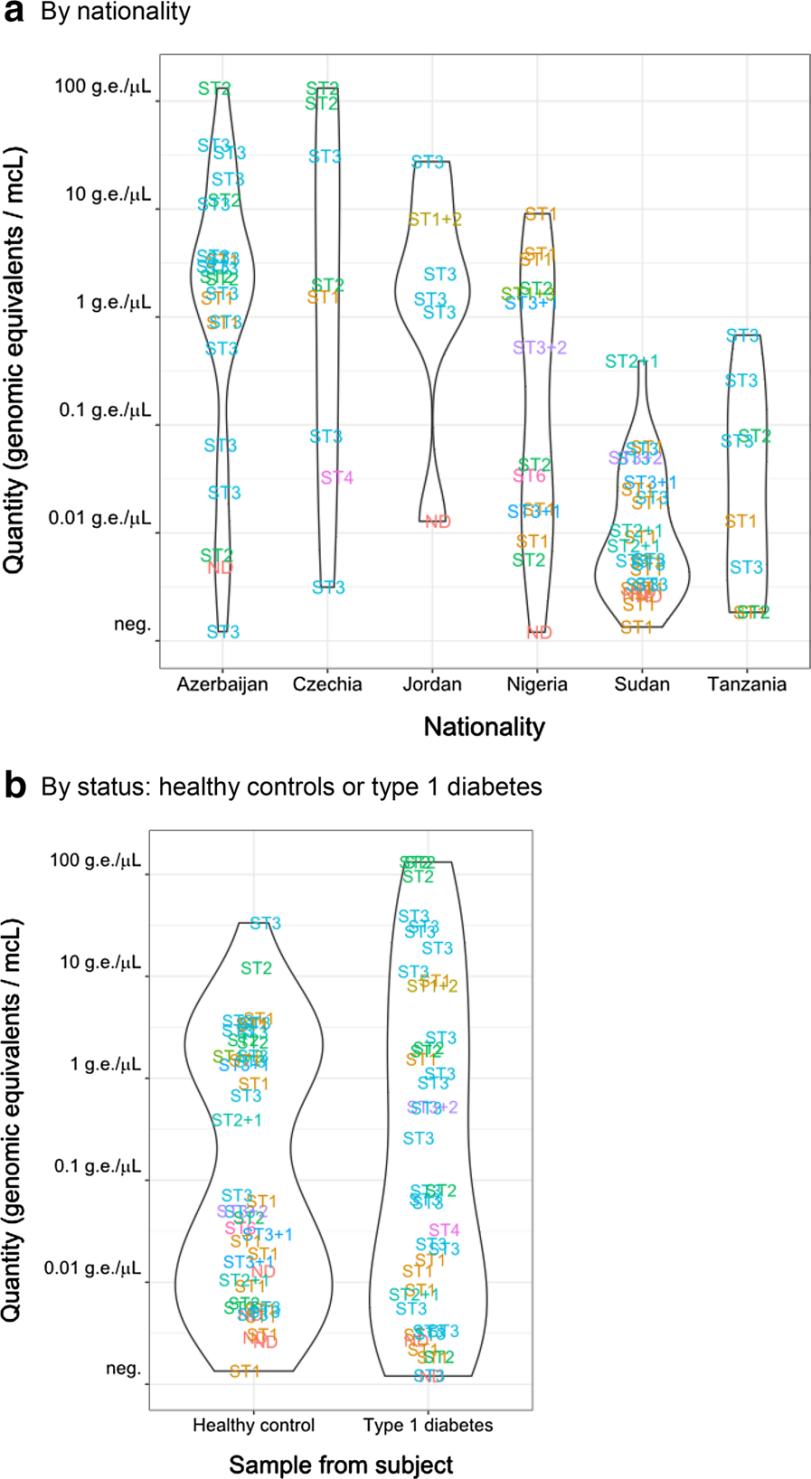
Quantity of *Blastocystis* in positive samples. The text points refer to the *Blastocystis* subtypes (or combination thereof) found in the samples. ND: subtype not determined

There was a clear difference in positivity rates between countries. The lowest positivity was noted in Jordan (15.0%) and Czechia (15.4%), significantly lower than in the remaining countries that had 45–55% samples positive (Table 2). The quantity of *Blastocystis* in positive samples also differed: Azerbaijan, Jordan and Czechia had higher quantities of *Blastocystis* than Nigeria, Sudan and Tanzania (Fig. 3A).

**Table 2:** *Blastocystis* positivity in specific PCR detection

	*Blastocystis* positivity *n*/*N*, (%)	OR for *Blastocystis* positivity^1^ OR [95% CI]
By country		
Azerbaijan	23/51 (45%)	1.0, reference
Czechia	8/52 (15%)	**0.19** [0.064–0.50], *p* = 0.0012
Jordan	6/40 (15%)	**0.21** [0.070–0.54], *p* = 0.0037
Nigeria	15/27 (55%)	1.50 [0.58–3.90]
Sudan	28/59 (47%)	1.15 [0.54–2.48]
Tanzania	8/15 (53%)	1.38 [0.42–4.58]
By diabetes status		
Type 1 diabetes	43/132 (32.6%)	1.26 [0.68–2.36]
Healthy controls	45/112 (40.2%)	1.0, reference
Total	88/244 (36.1%)	

^1^From a logistic regression model where dichotomized PCR positivity (positive at any level) was the outcome and the country of origin and type 1 diabetes (or healthy control) were two independent categorical predictors

The status of the subject (control child versus recently diagnosed patient with type 1 diabetes) influenced neither quantity (*p* = 0.28) nor positivity rate (*p* = 0.45) of *Blastocystis (*Fig. 3B). Similarly, there was no difference by gender (*p* = 0.43 for positivity and *p* = 0.69 for quantity).

### Blastocystis sequence types

Five distinct *Blastocystis* subtypes were identified. The ST3 was the most prevalent type (positive in 40/81, 49.4% sequenced samples), followed by ST1 (29/81, 35.8%) and ST2 (20/81, 24.8%), whereas ST4 and ST6 were each positive in one sample only (Table 3). The absolute quantity of *Blastocystis* did not differ by sequence type (*p* = 0.09), and the distribution of sequence types differed by neither nationality (*p* = 0.22) nor diabetes status (*p* = 0.22; Fig. 3).

**Table 3:** Sequence types of *Blastocystis* found in the six populations

	Samples sequenced, *n*	ST1 positive	ST2 positive	ST3 positive	ST4 positive	ST6 positive	Single ST *n* (%)	Two STs *n* (%)	Of double infections		
									ST1 + 2	ST1 + 3	ST2 + 3
By country											
Azerbaijan	22	3 (14%)	5 (23%)	14 (64%)	0	0	22 (100%)	0			
Czechia	8	1 (13%)	3 (38%)	3 (38%)	1 (25%)	0	8 (100%)	0			
Jordan	5	1 (20%)	1 (20%)	4 (80%)	0	0	4 (80%)	1 (20%)	1	0	0
Nigeria	14	8 (57%)	4 (29%)	5 (36%)	0	1 (7%)	10 (71%)	4 (29%)	0	3	1
Sudan	24	14 (58%)	5 (21%)	10 (42%)	0	0	19 (79%)	5 (21%)	3	1	1
Tanzania	8	2 (25%)	2 (25%)	4 (50%)	0	0	8 (100%)	0			
By diabetes status											
Type 1 diabetes	41	10 (24%)	11 (27%)	22 (54%)	1 (2.4%)	0	38 (93%)	3 (7.3%)	2	0	1
Healthy controls	40	19 (48%)	9 (23%)	18 (45%)	0	1 (2.5%)	33 (83%)	7 (18%)	2	4	1
Total (% of sequenced)	81	29/81 (35.8%)	20/81 (24.7%)	40/81 (49.4%)	1/81 (1.2%)	1/81 (1.2%)	71/81 (87.7%)	10/81 (12.3%)	4/81 (4.9%)	4/81 (4.9%)	2/81 (2.5%)

*n* number of samples; % of sequenced samples

Combined positivity for two different sequence types was observed in 10/81 (12.3%) *Blastocystis*-positive samples, whereas the remaining 87.7% were positive for a single sequence type. No sample was positive for three or more sequence types. Of the double-positive samples, combinations of ST1, 2 and 3 were noted, whereas the ST4 and ST6 were observed in a single sample each and alone.

The next-generation amplicon sequencing failed to determine the subtype of 7/88 (8%) of samples that had been found positive using real-time PCR. Of these untypable samples, three failed to produce a distinct PCR signal in the genotyping reaction, and four produced only off-target signals (mostly bacterial). All these failed samples had a very low *Blastocystis* quantity with Ct between 36.5 and 42.7 (Additional file 1: Figure S2).

### Intra-individual and intra-subtype variation

**Fig. 4:**
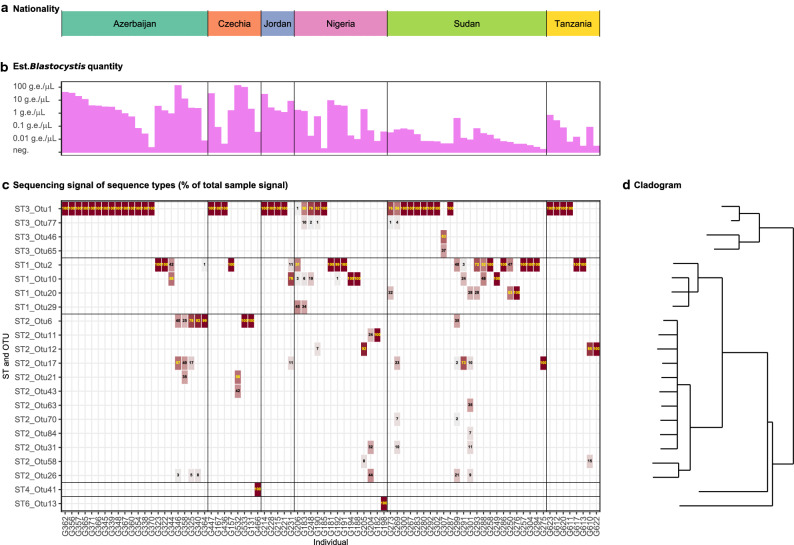
*Blastocystis* subtypes and intra-subtype variations analysed at a 98% similarity threshold

The PCR amplicons classified into 84 distinct zero-radius operational taxonomic units (ZOTUs) identified by USEARCH61. All ZOTUs were easily classifiable to the above listed existing sequence types (for phylogenetic tree, see Additional file 1: Figure S4). Upon similarity clustering at 97%, the count of distinct operational taxonomic units (OTUs) was reduced to 22; their distribution among samples along with sequence similarity is shown in Fig. 4. Of these OTUs, 8 were identical to sequences previously deposited to GenBank, 13 showed an identity of 97% or higher with previously deposited sequences, and 1 was 96% identical to previously deposited sequences (accession MW301904); neither of them was < 95% similar, which would have suggested a novel subtype (Additional file 1: Table S2). As to the intra-subtype composition, most individuals carried multiple ZOTUs, but after reduction by similarity to 97%, 56/81 samples could be characterized by a single OTU.

### Blastocystis and the faecal bacteriome

*Blastocystis* positivity, as well as its quantity, was associated with higher alpha diversity of the bacteriome (i.e. diversity within samples, Additional file 1: Table S3). This association was highly significant for the observed number of bacterial species, the Chao1, ACE, Fisher and Shannon indices in models adjusted for nationality. In contrast, Simpson index was not associated with *Blastocystis* presence or its quantity. Crude values of indices are plotted in Additional file 1: Figure S5.

**Fig. 5:**
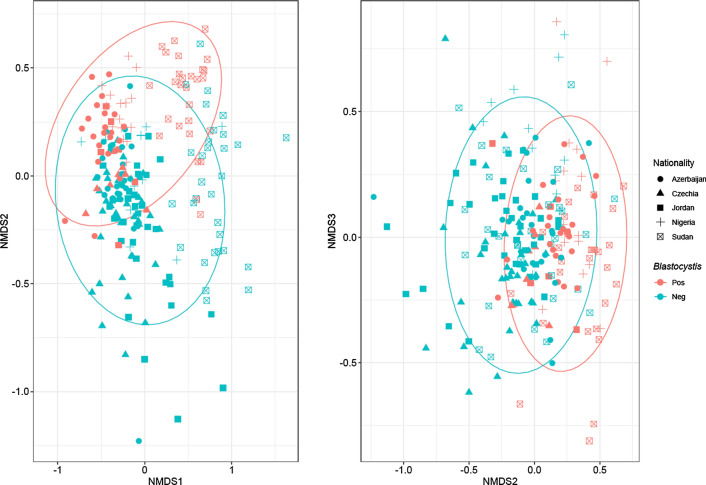
Bacteriome profiles grouped by *Blastocystis* positivity independent of their geographic origin. Non-metric multidimensional scaling of Bray-Curtis dissimilarity index, displayed by *Blastocystis* positivity and nationality. Three dimensions were used for NMDS, as the third dimension improved the fit of ordination. Shape shows nationality; color shows PCR positivity for *Blastocystis*. *Blastocystis* positivity affects position of the samples along NMDS axis 2

The beta (between samples) diversity of the bacteriome was expressed by Bray-Curtis distance on double-Wisconsin transformed data agglomerated at the taxonomic level of genus. Ordination was performed using NMDS (Fig. 5). The final ordination had three dimensions, because adding the third dimension considerably decreased the stress and improved the fit (non-metric fit between observed dissimilarity and ordination distance had *R*^2^ = 0.987 with stress of 0.116). The *Blastocystis* positivity projected along the second axis, whereas the sample origin associated with the first axis, with a separation of the Sudanese samples from the rest of the dataset.

The PERMANOVA testing of group mean differences of beta diversity showed that *Blastocystis* positivity (*p* < 0.001) and nationality of the subject (*p* < 0.001) were independently associated with differences in the bacteriome composition at the genus level, and the significant interaction term (*p* = 0.010) showed that the strength of this association varied among nationalities. The *Blastocystis* positivity explained 2.0% of the total bacteriome variance, and the interaction with the nation of origin explained a further 2.1%. When constructing the model at other taxonomic levels than genus, the *Blastocystis* term was highly significant from the high-resolution level of bacterial amplicon sequencing variants up to the level of class, but not at the phylum level. All three major subtypes (ST1, ST2, ST3) significantly affected the bacteriome composition if tested in the PERMANOVA model as separate terms, with no significant mutual interactions (data not shown). Models with logarithm quantity of *Blastocystis* as the independent variable (instead of dichotomous positivity) showed analogous associations (data not shown). Essentially identical highly significant association signal was obtained also when the PERMANOVA permutations were restricted within the tested nationalities (i.e. as if the design had blocks at the nationality level). The respective terms in the PERMDISP tests of homogeneity of multivariate variances were all statistically insignificant, the variances did not differ in any of the compared nationality-positivity pairs (data not shown), which indicated that the above associations observed in PERMANOVA were due to genuine differences between the position of *Blastocystis*-positive and *Blastocystis*-negative centroids rather than due to differences in spread.

We then tested *Blastocystis* association with bacteriome composition using constrained ordination, the redundancy analysis on the Hellinger-transformed abundance data. We arrived at analogous results: both nationality and *Blastocystis* were significant predictors (*p* < 0.001). *Blastocystis* positivity explained 3.0% of the total variance in bacteriome composition, and an additional small proportion of the total variance was explained by interactions between nationality and *Blastocystis* positivity (again indicating a significant heterogeneity in the effect between nationalities). The results remained materially unchanged when the three major subtypes (ST1, ST2, ST3) were entered in the model as separate positivity terms (data not shown).

Finally, individual taxa were tested for association with *Blastocystis* positivity. The *DESeq2* tests revealed several signals: *Bifidobacterium, Blautia, Subdoligranulum* and several less abundant genera were decreased in *Blastocystis*-positive samples, whereas *Ruminococcaceae UCG-002* and *Ruminococcaceae UCG-014* were positively associated with *Blastocystis* (Table 4; Additional file 1: Figure S6). The signals partly propagated upwards into the level of family (inverse association of *Blastocystis* with *Bifidobacteriaceae, Streptococcaceae* and *Lactobacillaceae*), order (*Bifidobacteriales* and *Lactobacillales*), class (*Actinobacteria*) and phylum (*Actinobacteria*). The differences were of magnitudes within the range of 0.22–4.16. All models were adjusted for nationality; most differences were also appreciable upon visual inspection of nationality-stratified abundance graphs (Additional file 1: Figure S7).

**Table 4:** Significant signals from *DESeq2* testing of association between *Blastocystis* positivity and individual taxa within the stool bacteriome

Phylum	Class	Order	Family	Genus	Mean abundance of genus	Fold-diff (genus)	Corrected adjusted *p* value
***↓* Actinobacteria**	***↓* Actinobacteria**	***↓* Bifidobacteriales**	***↓* Bifidobacteriaceae**	***↓ Bifidobacterium***	6.0%	0.36	6.2 × 10^–6^
Firmicutes	Negativicutes	Selenomonadales	Veillonellaceae	***↓ Veillonella***	1.3%	0.22	3.0 × 10^–4^
	Bacilli	***↓* Lactobacillales**	***↓* Streptococcaceae**	***↓ Streptococcus***	1.5%	0.28	9.4 × 10^–7^
			***↓* Lactobacillaceae**	***↓ Lactobacillus***	1.3%	0.39	7.7 × 10^–3^
	Clostridia	Clostridiales	Lachnospiraceae	***↓ Fusicatenibacter***	1.6%	0.45	1.0 × 10^–3^
				***↓ Blautia***	7.9%	0.42	8.9 × 10^–8^
				***↓ Anaerostipes***	1.6%	0.38	6.2 × 10^–6^
			Ruminococcaceae	***↓ Subdoligranulum***	2.3%	0.48	7.9 × 10^–4^
				***↑ Ruminococcaceae UCG-002***	1.5%	2.65	8.1 × 10^–5^
				***↑ Ruminococcaceae UCG-014***	1.6%	4.16	7.7 × 10^–4^

All models were adjusted for nationality; PCR positivity for *Blastocystis* was a dichotomous predictor

The model predictors were nationality and *Blastocystis* positivity in real-time PCR. The fold difference is the coefficient for the particular genus, associated with *Blastocystis* positivity. The *P* value comes from this adjusted *DESeq2* model and is corrected for the number of tested taxa. Shown are significantly associated taxa with a mean abundance > 1%

Arrows and bold typeface indicate statistically significant associations

## Discussion

In the present study, we quantified and subtyped *Blastocystis* in sample sets from children of six distant countries and found that *Blastocystis* was associated with both an increased alpha diversity of faecal bacteriome and its community composition. We noted marked differences in *Blastocystis* frequency by country, but no significant difference were observed between children with recent-onset diabetes versus matched control subjects.

### Blastocystis prevalence and quantity

Using parasitome profiling, we first confirmed that *Blastocystis* was the most abundant gut protist in children across the studied populations. Then, we utilized specific quantitative real-time PCR for the detection and accurate quantification of the organism. The prevalence varied among countries: Czechia and Jordan had significantly lower prevalence than the remaining populations. Also the quantity distribution of *Blastocystis* differed: it appears that samples from Sudan and Tanzania were predominantly of lower quantity, whereas quantities in Jordan, Azerbaijan and Czechia had a bimodal distribution with an accent on higher quantities. Nigerian samples contained a continuum of quantities. Conceivably, this might reflect the stages of infection: higher quantities may be due to acute replication in contrast to low quantities reflecting long-term established carrier status.

It is difficult to compare our positivity rates to those from previous studies—there has been an enormous heterogeneity in the inclusion criteria and also in detection strategies, resulting in the widest possible spread of published prevalence: from 30 of 6422 (0.5%) Japanese subjects, using microscopy [26], to 100% of 93 Senegalese children tested by PCR [27]. Several studies should be however pinpointed that performed *Blastocystis* detection and typing in the presently reported populations. Petrášová et al. reported data from six researchers working at a Tanzanian primate forest reserve (all 6 were positive) [28]; Lhotská et al. studied volunteers in Czechia, finding a 24.3% (12/49) positivity in children [29], not significantly different from the present report; Alfellani et al. studied a rather heterogeneous collection of datasets that also included Nigerian patients (adults, with 49% positivity) [9]. Finally, rural children in Nigeria were studied by Poulsen et al. [30] who reported a prevalence of 84% in the village of Illero in Western Nigeria. This is significantly more than the 55% we observed from our sample collected in the city of Abakaliki and environs. The reasons for this difference may be many-fold, from the difference in age composition (children in Illero were younger than our group) to the size of the community and ensuing differences in water supply, sanitation and lifestyle. It has been hypothesized that differences in *Blastocystis* prevalence among countries are primarily attributable to the standards of hygiene, especially of food and water, and to the exposure to animals (e.g. [2]). We cannot directly test these predictors, as we lack relevant metadata. However, our children originate mostly from urban and peri-urban areas, which may partly explain the lack of profound differences according to geographic origin: the subjects' hygienic conditions may in fact be more similar across the nationalities than would be between urban and remote rural populations.

### Blastocystis subtypes

The ST3 was the most often detected subtype among Azerbaijani, Jordanian and Tanzanian children, in accord with its earlier reported worldwide predominance [9]. Interestingly, Nigeria has previously reported slightly more ST1 than ST3 in subjects from the metropolitan area of Lagos [9], and the present study in children from Abakaliki and environs seems to confirm this. This holds also for Sudanese children. ST4 was detected only once: this sole occurrence was from Czechia, in accord with the European predilection of ST4 [3]. The paucity of ST4 in our asymptomatic sample set is in accord with the reported association of ST4 with symptomatic infections [31]: in the present study, diarrhoea or other acute signs of infection were exclusion criteria. Finally, ST6, a subtype whose common source is birds [32, 33], was noted only in one sample from Nigeria.

Co-infections with two different subtypes were not exceptional, totalling to 12.3% of positives. We believe that co-infections by several strains of the same subtype were also frequent: these are more difficult to detect, as the amplicon diversity captures both intra-organismal differences among genomic copies of 18S rDNA and intra-subtype differences between strains. Intra-organismal diversity among 18S rDNA copies was convincingly demonstrated already in 2012 when Meloni et al. sequenced 50 subclones of 18S rDNA amplicons from a woman co-infected with three STs [34]. Also our amplicon NGS provides ample indirect indications of such intra-organismal diversity and also of co-infections with more strains of the same subtype (Additional file 1: Figure S4), but a definite proof by subculturing could not be provided from our long-term frozen samples.

### *Blastocystis* and the bacteriome

We observed a clear direct association of *Blastocystis* positivity or quantity with higher richness of the bacteriome. Such an increased alpha diversity in *Blastocystis*-positive stool samples has been repeatedly reported from previous works (e.g. [10]]). We observed no association with the Simpson index, which captures evenness on top of richness; similarly, Audebert et al. [35] found the difference in alpha diversity notably more pronounced in the Shannon than Simpson index. It may be speculated that *Blastocystis* thrives on certain bacteria that are present only in rich communities or that it is the richness itself which makes a suitable environment for the protist. Alternatively, the protist itself might modify the bacterial community composition in accord with its presumed role of an 'ecosystem engineer' [36].

Positivity of *Blastocystis* was also associated with a difference in the bacterial community composition. Although only little of the beta (between-samples) diversity was explained by *Blastocystis*, the association was highly statistically significant and was present consistently across the nationalities, albeit at variable strength. It also appears that any of the three major observed subtypes (ST1, ST2 and ST3) associates with the bacteriome in the same direction, i.e. that it is the fact of positivity for *Blastocystis* rather than its particular subtype that matters.

We attempted to find individual taxonomic units associated with *Blastocystis* positivity. Many genera were mildly associated in either direction. This suggests a complex relationship (e.g. dependence on metabolic products or other phenotyping bacterial traits) or a potential unexplored confounder affecting both the bacteriome and *Blastocystis.*

Previously published association studies on *Blastocystis* and the bacterial community composition are notably heterogeneous, yet several papers reported a decreased abundance of members of Bacteroidales, namely *Bacteroides* or *Prevotella*. In a retrospective analysis of stool metagenomes, Andersen et al. [10] found an inverse association with an enterotype dominated by *Bacteroides* (see below for enterotypes and their lack of association in the present study). A similar author team revisited the topic a year later [37], using real-time PCR quantification of six candidate taxa: *Bacteroides* was again negatively associated with *Blastocystis*, and this inverse association was further enhanced by *Dientamoeba* positivity. Importantly, such an association was detected not only in healthy subjects, but also in patients with intestinal symptoms. *Bacteroides*-driven enterotypes were negatively associated with *Blastocystis* also in a large study from the Flemish Gut Flora Project [4]. Higher bacterial richness and lower abundance of *Bacteroides* with *Blastocystis* positivity were noted also in a modestly sized study of Swedish travelling students [38], along with several weaker association signals from other taxa, and similar indications on alpha diversity and inverse association with *Bacteroides* originated from subgroup analyses in a study of irritable bowel syndrome [39]. An important large study was the reanalysis of 12 multi-national metagenomic shotgun sequencing datasets of healthy, obese and diseased subjects by Beghini et al. [7]. They identified both positive associations with *Blastocystis* (for e.g. *Buryrivibrio, Prevotella, Akkermansia, Bifidobacterium*) and inverse associations for multiple *Bacteroides* species (significant in 5 of the 12 analysed datasets). Gabrielli et al. [40] performed bacteriome analysis in a modestly sized group of 18 ST3-positive and 9 *Blastocystis*-negative younger males of various primary diagnoses, observing not only a higher bacteriome diversity associated *Blastocystis* positivity, but also a profound imbalance within the Bacteroidales class where *Blastocystis* carriers had less Bacteroidaceae and more Prevotellaceae. Several other therein reported differences would not survive the corrections for the number of performed tests. Associations of numerous taxa were identified by Audebert et al. [35] at the levels of class, order, family and genus, mostly disjunct with the groups identified by the present study: of note, inverse association with *Blastocystis* was observed for *Prevotella* but not *Bacteroides.*

In contrast to the above reports, our mutinational dataset showed no indication of *Blastocystis* association with either *Bacteroides* or *Prevotella.* Of note, our populations were very heterogeneous in the abundance of the two genera—this was expected, as both serve as markers for westernized versus non-westernized lifestyle, e.g. [41]. It is therefore conceivable that in our negative binomial models with the country of origin as one of the terms, an isolated association in one or two populations would not lead to the overall statistical significance.

*Bifidobacterium* was inversely associated with *Blastocystis*, having in average three-fold lower abundance in *Blastocystis*-positive samples. This is in accord with the results by Beghini et al. [7] and also by Nourrisson et al. [42] who observed in a subgroup analysis of healthy males that *Blastocystis* was associated with a decrease in *Bifidobacterium* (the same direction as we observed) and an increase in *Lactobacillus* (i.e. the opposite direction to the present study). *Bifidobacterium* is an important genus whose aproximately ten species inhabit the human gut from early infancy to an advanced age. Its main functions include stabilization of the early bacteriome, preserving intestinal barrier, immune education and the switch between infant and childhood microbiome [43, 44]. The positive perception of its function, including its use as probiotic [45], is in contrast to the here observed inverse association with *Blastocystis*, an organism now gradually recognized as beneficial to the gut health (e.g. [46]). Interestingly, in a mouse model, a pathogenic ST7 decreased the abundance of two taxa beneficial for gut health, *Bifidobacterium* sp. and *Lactobacillus* sp. [47], both of whom were inversely associated with *Blastocystis* also in the present study. Clearly, more studies are warranted, optimally using longitudinal sampling to enable causative inference.

Studies are scarce that investigate subjects outside Europe and the US for the association between *Blastocystis* and the bacteriome. A small preliminary study published from Côte d'Ivoire used older methods not readily comparable to massively parallel 16S rDNA profiling [48]; authors' interpretation suggested that *Entamoeba* and *Blastocystis* may shift the microbiota balance towards eubiosis. A study on 156 asymptomatic adults from semi-industrialised rural Mexico demonstrated an increased alpha diversity in *Blastocystis*-positive individuals, and suggested numerous differences in bacterial abundance—most prominent of these being lower *Prevotella* (class Bacteroidia, order Bacteroidales) but not *Bacteroides*, similarly to what was found by Audebert et al. [35]. Two concurrently published modestly sized studies from schoolchildren living in Colombia both confirmed the increased richness of bacteriomes, and one of them also found *Prevotella* more abundant in samples with *Blastocystis* [49, 50]. A study on 296 young healthy children from Mali reported that *Blastocystis* colonized children had an increased bacterial richness and different community structure in ordination [51] as well as an unusually high number of differentially abundant bacterial taxa—this might partly be attributable to the choice of significance threshold and to bioinformatic analyses.

Several of the above studies suggested that *Blastocystis* may associate with distinct enterotypes, and also Andersen et al. [10] retrospectively analysed metagenomic data from 316 stool samples, finding *Blastocystis* significantly less often in the *Bacteroides*-driven enterotype than in the *Prevotella-* or *Ruminococus*-driven enterotypes. Enterotypes are entities simplifying the composition of the bacteriome into a few categories. Others and we are of the opinion that enterotypes are a rather controversial concept, because of oversimplification and lack of generalizability, especially in a dataset like ours that consists of multiple distant population samples. To test for association with enterotypes, we revisited our earlier performed Dirichlet classification modelling of the present bacteriome data [12]. Neither of these enterotypes associated with *Blastocystis* positivity (data not shown). It should be however noted that the composition of our three components was much less clear-cut than in the dataset reported by Andersen et al. [10], probably because of the international character of our sample set.

### Strengths of the study

Our study provides a direct comparison among multiple sample sets, using identical inclusion criteria and identical protocols for collection, storage and handling of samples. The laboratory analysis was centralized. Separate reactions were used for the detection of *Blastocystis* and for its classification into sequence types (subtyping). Because the subtyping primers are flanked with adaptors that enable subsequent addition of indices and sequencing anchors, such a PCR reaction must be—at least in theory—less efficient than a PCR with shorter plain primers. Therefore, we first utilized the highly sensitive and specific real-time PCR with a hydrolysis probe for detection and quantification, and then the typing reaction was run separately. The correctness of this approach is documented by the existence of 8% samples that contained quantifiable amounts of *Blastocystis*, yet failed to yield a subtype. Expectedly, real-time PCR was superior in sensitivity and specificity also to profiling of eukaryotic microorganisms that we used for the initial screening (Additional file 1: Figure S8).

We reported not only positivity, but also *Blastocystis* quantity and used it in analyses of subtypes as well as of association with the bacteriome composition. Although absolute quantification from stool is cumbersome (there is no internal standard for reference), we believe that this quantitative aspect reinforced especially the finding of association with the bacteriome.

In *Blastocystis* subtyping, we first analysed the raw ZOTUs (i.e. observed sequences with zero sequence similarity) and only then agglomerated the reads by similarity. The fine-grained resolution of ZOTUs helped define the intrinsic variability of *Blastocystis* 16S rDNA amplicons. This variability consists of intra-organismal variation between individual ribosomal DNA genes, variations between strains and co-infection with different strains of the same subtype. Moreover, the degree of variation among strains of the same subtype probably differs between subtypes [52]. Although we did not subculture the stools with presumed co-infection, we can indirectly point to patterns of ZOTUs emerging from the detailed variant mapping (Additional file 1: Figure S4).

We tested several approaches that characterize the ST from massive parallel amplicon sequencing data. For an assignment of subtypes from a closed reference set, our previously published tool VIPIE was a rapid and reliable alternative [53]—we however wanted to capture all variants, including potential distant novel subtypes, so we instead relied on the de-novo identified ZOTUs by USEARCH61. Of note, not all bioinformatic pipelines tailored for bacteriome research may be are equally well suited for characterizing the *Blastocystis* amplicon variants: for instance, the DADA2 pipeline [23] erroneously eliminated the ST6 amplicon sequence variant from our dataset, since it was present only in one sample and probably resembled a sequencing chimera artefact (data not shown).

### Limitations and their remedies

The first limitation is linked to unavailability of a fresh sample. Only DNA analyses were performed—the use of frozen stool precluded in vitro culture or direct morphological assessment by microscopy. We do not know what stages (forms) of *Blastocystis* were present and whether morphology related to quantity, genetic subtypes or sample origin. We also could not perform subcultures assessing the intraorganismal variance of the 18S rDNA.

Another limitation may be the lack of longitudinal samples; only one specimen was collected from each individual. Although consecutive samples in short time intervals reportedly do not increase the detection rate using concentration and microscopy [54], this may not be the case for PCR. Moreover, revisiting subjects in a longer time interval, e.g. after a year, and subtyping their *Blastocystis* would demonstrate the stability of colonization and the persistence of strains. However, the logistics of repeated sampling of the same subjects proved impracticable in the multinational setting with our limited budget. Causality cannot be inferred from a cross-sectional study like ours, so it remains unresolved whether *Blastocystis* is an active modifier of the bacteriome content (e.g. by putative phagocytic properties directed towards overgrowing species) or whether *Blastocystis* is a marker organism thriving on certain rare species or on a higher degree of bacterial diversity. The association may also be confounded by a factor yet undefined and unmeasured, e.g. the passage time, or the proportion of material from mucosal surfaces versus luminal flora.

Lastly, the primary purpose for collecting the sample set was studying type 1 diabetes; many subjects were thus recently diagnosed with this condition. *Blastocystis* was not associated with diabetes in either of the populations, so we believe that this was not a significant source of bias. Notably, use of sample sets originally collected for other purposes is common to most published studies on bacteriomes and *Blastocystis*—many collections originated from studies of IBS, IBD, ulcerative colitis, diarrhoea, obesity, type 2 diabetes and others.

## Conclusions

*Blastocystis* is the most frequent component of intestinal protist fauna in asymptomatic children, and it is not exceptional to carry multiple distinct subtypes or several variants of the same subtype. *Blastocystis* is linked to both an increased faecal bacteriome alpha diversity and its composition, although no clear-cut *Blastocystis*-associated patterns were detected across the multi-national sample set.

## Supplementary Information

**Additional file 1: Table S1.** Parasites identified in molecular survey by massively parallel sequencing. **Table S2.** Operational taxonomic units (OTUs) of Blastocystis amplicon subtyping in our study. **Table S3.** Alpha diversity of bacteriome is associated with the positivity for Blastocystis. **Table S4.** Results of PERMANOVA tests at different taxonomic levels. **Table S5.** Association of Blastocystis positivity with the bacterial taxa: results of DESeq2 tests with relaxed filtering by abundance. **Figure S1.** Screening of eukaryotic parasites in stool using 18S rDNA profiling. **Figure S2.** Genotyping reactions, with the flowchart of failed reactions. **Figure S3.** Blastocystis subtypes and intra-subtype variations without reducing the number of ZOTUs. **Figure S4.** Phylogram of all ZOTUs in the study on the background of GenBank sequences representative of Blastocystis subtypes. **Figure S5.** Alpha diversity of the bacteriome by positivity for Blastocystis. **Figure S6.** Heatmap showing the relative abundance of bacterial genera associated with Blastocystis positivity. **Figure S7.** Abundance of bacterial genera associated with Blastocystis positivity. **Figure S8.** Comparison of specific real-time PCR for Blastocystis to the signals from the profiling of eukaryotic microorganisms. **Supplementary Methods.** Massively parallel 18S rDNA profiling of the eukaryotic parasitome.

## Data Availability

The datasets supporting the conclusions of this article are available in the NCBI Sequencing Read Archive (SRA): the raw *Blastocystis* amplicon sequencing data and the metadata of individual positive samples have been uploaded under project accession number PRJNA681600, the sequence data from bacteriome profiling under accession PRJNA445932. The sequences of operational taxonomic units (OTUs) of *Blastocystis* have been deposited to the GenBank database under accession numbers MW301893-MW301914 (Additional file 1: Table S1).
