# Supplementary material for: Blastocystis in the faeces of children from six distant countries: prevalence, quantity, subtypes and the relation to the gut bacteriome
Source: Parasit Vectors. 2021 Aug 12;14:399. doi: 10.1186/s13071-021-04859-3 (PMC8359624; doi:10.1186/s13071-021-04859-3)
Supplement: Supplementary file 1 — Additional file 1: Table S1. Parasites identified in molecular survey by massively parallel sequencing. Table S2. Operational taxonomic units (OTUs) of Blastocystis amplicon subtyping in our study. Table S3. Alpha diversity of bacteriome is associated with the positivity for Blastocystis. Table S4. Results of PERMANOVA tests at different taxonomic levels. Table S5. Association of Blastocystis positivity with the bacterial taxa: results of DESeq2 tests with relaxed filtering by abundance. Figure S1. Screening of eukaryotic parasites in stool using 18S rDNA profiling. Figure S2. Genotyping reactions, with the flowchart of failed reactions. Figure S3. Blastocystis subtypes and intra-subtype variations without reducing the number of ZOTUs. Figure S4. Phylogram of all ZOTUs in the study on the background of GenBank sequences representative of Blastocystis subtypes. Figure S5. Alpha diversity of the bacteriome by positivity for Blastocystis. Figure S6. Heatmap showing the relative abundance of bacterial genera associated with Blastocystis positivity. Figure S7. Abundance of bacterial genera associated with Blastocystis positivity. Figure S8. Comparison of specific real-time PCR for Blastocystis to the signals from the profiling of eukaryotic microorganisms. Supplementary Methods. Massively parallel 18S rDNA profiling of the eukaryotic parasitome. [file 13071_2021_4859_MOESM1_ESM.pdf]

Supplementary material to

Blastocystis in the faeces of children from six distant countries: prevalence, quantity, subtypes and the relation to the gut bacteriome

Table S1. Parasites identified in molecular survey by massively parallel sequencing

	**Samples screened**	***Blastocystis*** n positive (%)	***Entamoeba*** n positive (%)	***Dientamoeba*** n positive (%)	***Endolimax*** n positive (%)
Azerbaijan	51	24 (47%)	1 (2.0%)	6 (12%)	1 (2.0%)
Czechia	52	8 (15%)	0	3 (5.8%)	0
Jordan	40	8 (20%)	1 (2.5%)	2 (5.0%)	0
Nigeria	27	13 (48%)	5 (19%)	1 (3.7%)	1 (3.7%)
Sudan	59	20 (34%)	6 (10%)	1 (1.7%)	6 (10%)
Tanzania	15	4 (27%)	3 (20%)	0	0
**Total**	**244**	**77 (32%)**	16 (6.6%)	13 (5.3%)	8 (3.3%)

Table S2. **Operational taxonomic units (OTUs) of *Blastocystis* amplicon subtyping in our study**

OTU	Sequence type	Count of samples positive for the allele (positivity as % of *Blastocystis* amplicon signal)						GenBank			PubMLST allele		
		neg	0.05% - 1%	1.1% - 10%	11%- 50%	51% - 90%	>90%	GenBank Accession of this OTU	Closest previously reported sequence in GenBank	Identity to the closest previously reported sequence	Closest PubMLST allele	Bases different to this PubMLST allele	Count of bases different to this allele
Otu1	ST3	42	1	0	0	4	34	MW301893	e.g. MT186210.1	100,00%	34	0 (0,0%)	0
Otu2	ST1	59	1	1	4	3	13	MW301894	e.g. KX524008.1	100,00%	88	0 (0,0%)	0
Otu6	ST2	73	0	0	3	1	4	MW301895	e.g. MT089925.1	100,00%	13	1 (0,2%)	1
Otu10	ST1	70	0	3	3	2	3	MW301896	KU147350.1	100,00%	2	11 (2,4%)	11
Otu12	ST2	77	0	1	0	1	2	MW301897	MK801368.1	97,55%	13	14 (3,1%)	14
Otu17	ST2	72	0	2	4	2	1	MW301898	HQ641605.1	99,33%	9	7 (1,6%)	7
Otu20	ST1	76	0	0	3	1	1	MW301899	MT903376.1	98,89%	2	15 (3,3%)	15
Otu11	ST2	79	0	0	1	0	1	MW301900	MK874805.1	97,55%	13	13 (2,9%)	13
Otu41	ST4	80	0	0	0	0	1	MW301901	e.g. MK587493.1	100%	42	0 (0,0%)	0
Otu13	ST6	80	0	0	0	0	1	MW301902	MN472769.1	100%	122	2 (0,5%)	2
Otu21	ST2	79	0	0	1	1	0	MW301903	MK874793.1	100,00%	9	8 (1,8%)	8
Otu46	ST3	80	0	0	0	1	0	MW301904	HQ909891.1	96,05%	34	30 (6,6%)	30
Otu26	ST2	75	0	4	2	0	0	MW301905	MK874814.1	99,33%	13	19 (4,2%)	19
Otu31	ST2	78	0	1	2	0	0	MW301906	MK874805.1	99,33%	13	9 (2,0%)	9
Otu29	ST1	79	0	0	2	0	0	MW301907	MK874819.1	99,56%	2	12 (2,7%)	12
Otu77	ST3	76	0	4	1	0	0	MW301908	MK874800.1	97,80%	34	15 (3,3%)	15
Otu58	ST2	79	0	1	1	0	0	MW301909	MK874811.1	100,00%	13	23 (5,1%)	23
Otu43	ST2	80	0	0	1	0	0	MW301910	MK874805.1	99,78%	9	7 (1,6%)	7
Otu63	ST2	80	0	0	1	0	0	MW301911	MK874793.1	99,33%	13	8 (1,8%)	8
Otu65	ST3	80	0	0	1	0	0	MW301912	HQ909891.1	99,56%	34	31 (6,9%)	31
Otu70	ST2	79	0	2	0	0	0	MW301913	MK874793.1	99,11%	13	7 (1,6%)	7
Otu84	ST2	80	0	1	0	0	0	MW301914	MK874803.1	99,55%	9	8 (1,8%)	8

The OTUs are sorted by their overall positivity in the study.

Table S3. Alpha diversity of bacteriome is associated with the positivity for *Blastocystis*

**Alpha diversity index**	**Likelihood of *Blastocystis* positivity associated with a unit increase in diversity index (conditional logistic regression, conditioned for nationality)**	**Difference in diversity index associated with *Blastocystis* positivity (linear model adjusted for nationality)**	**Difference in diversity index associated with decadic log increase in *Blastocystis* quantity (linear model adjusted for nationality)**
Observed bacterial OTUs	1.021 [1.014 to 1.028], p = 3.98e-09	59.6 [43.5 to 75.7], p = 5.8e-12	24.1 [18.6 to 29.6], p = 1.1e-15
Chao1	1.016 [1.011 to 1.022], p = 8.67e-09	70.7 [51.2 to 90.1], p = 1.4e-11	28.7 [22.1 to 35.3], p = 2.5e-15
ACE	1.016 [1.010 to 1.021], p = 1.35e-08	68.7 [49.3 to 88.2], p = 3.9e-11	27.852 [21.2 to 34.4], p = 1.4e-14
Fisher	1.095 [1.062 to 1.129], p = 5.65e-09	13.0 [9.5 to 16.6], p = 7.6e-12	5.4 [4.2 to 6.6], p = 2.0e-16
Shannon	1.823 [1.091 to 3.047], p = 0.0229	0.211 [0.036 to 0.386], p = 0.019	0.111 [0.050 to 0.172], p = 4.4e-04
Simpson	1.671 [0.076 to 36.6], p = 0.75	0.005 [-0.022 to 0.031], p = 0.74	0.008 [-0.002 to 0.017], p = 0.11

Coefficients are shown with 95% confidence intervals, and the P value of the respective term.

Table S4. Results of PERMANOVA tests at different taxonomic levels

PERMANOVA was run with 1000 permutations, with composition of the bacteriome at the given level as the dependent variable. Bray-Curtis dissimilarity was used as the distance metric. The predictors were *Blastocystis* positivity, nationality, and their interaction.

**The level of taxonomic resolution**	**P (pseudo F test) of *Blastocystis* PCR positivity**	**P (pseudo F test) of nationality**	**P (pseudo F test) of interaction**	**R^2 of *Blastocystis* PCR positivity**	**R^2 of nationality**	**R^2 of their interaction**
Level of amplicon sequencing variants (original operational taxonomic units)	< 0.001	< 0.001	0.013	0.015	0.168	0.021
Level of genus	< 0.001	< 0.001	0.018	0.020	0.252	0.021
Level of family	< 0.001	< 0.001	0.007	0.016	0.304	0.023
Level of class	0.005	< 0.001	0.019	0.013	0.350	0.019
Level of phylum	0.067, NS	< 0.001	0.890	0.008	0.338	0.006

Table S5. Association of *Blastocystis* positivity with the bacterial taxa: results of *DESeq2* tests with relaxed filtering by abundance

Phylum	Class	Order	Family	Genus	Mean proportion per sample[1]	Fold-difference[2]	Corrected adjusted P value[2]
Actinobacteria	-	-	-	-	7,0%	0,45	6,4E-06
Actinobacteria	Actinobacteria	-	-	-	5,7%	0,31	2,3E-09
Actinobacteria	Actinobacteria	Bifidobacteriales	-	-	5,6%	0,36	7,1E-06
Actinobacteria	Actinobacteria	Bifidobacteriales	Bifidobacteriaceae	-	5,6%	0,34	1,3E-06
Actinobacteria	Actinobacteria	Bifidobacteriales	Bifidobacteriaceae	*Bifidobacterium*	6,0%	0,36	6,2E-06
Actinobacteria	Coriobacteriia	Coriobacteriales	Coriobacteriaceae	-	0,9%	0,43	4,6E-04
Actinobacteria	Coriobacteriia	Coriobacteriales	Coriobacteriaceae	*Collinsella*	0,9%	0,47	2,0E-03
Bacteroidetes	Bacteroidia	Bacteroidales	Rikenellaceae	*Alistipes*	0,7%	0,50	1,6E-02
Firmicutes	Bacilli	Lactobacillales	-	-	8,5%	0,47	8,3E-05
Firmicutes	Bacilli	Lactobacillales	Lactobacillaceae	-	2,4%	0,35	9,3E-04
Firmicutes	Bacilli	Lactobacillales	Lactobacillaceae	*Lactobacillus*	1,3%	0,39	7,7E-03
Firmicutes	Bacilli	Lactobacillales	Streptococcaceae	-	1,4%	0,28	4,9E-07
Firmicutes	Bacilli	Lactobacillales	Streptococcaceae	*Streptococcus*	1,5%	0,28	9,4E-07
Firmicutes	Clostridia	Clostridiales	Family_XIII	-	0,3%	2,13	2,3E-04
Firmicutes	Clostridia	Clostridiales	Family_XIII	*Family_XIII_AD3011_group*	0,1%	2,01	1,5E-03
Firmicutes	Clostridia	Clostridiales	Christensenellaceae	-	0,9%	2,13	3,9E-03
Firmicutes	Clostridia	Clostridiales	Christensenellaceae	*Christensenellaceae_R-7_group*	0,8%	2,20	2,0E-03
Firmicutes	Clostridia	Clostridiales	Lachnospiraceae	*Anaerostipes*	1,6%	0,38	6,2E-06
Firmicutes	Clostridia	Clostridiales	Lachnospiraceae	*Blautia*	7,9%	0,42	8,9E-08
Firmicutes	Clostridia	Clostridiales	Lachnospiraceae	*Coprococcus_2*	0,4%	2,19	3,3E-02
Firmicutes	Clostridia	Clostridiales	Lachnospiraceae	*Coprococcus_3*	0,4%	0,41	2,7E-03
Firmicutes	Clostridia	Clostridiales	Lachnospiraceae	*Fusicatenibacter*	1,6%	0,45	1,0E-03
Firmicutes	Clostridia	Clostridiales	Lachnospiraceae	*Lachnospiraceae_ND3007_group*	0,2%	2,53	8,8E-04
Firmicutes	Clostridia	Clostridiales	Ruminococcaceae	*Ruminococcaceae_NK4A214_group*	0,3%	3,43	8,7E-06
Firmicutes	Clostridia	Clostridiales	Ruminococcaceae	*Ruminococcaceae_UCG-002*	1,5%	2,65	8,1E-05

Firmicutes	Clostridia	Clostridiales	Ruminococcaceae	*Ruminococcaceae_UCG-005*	0,4%	3,69	2,0E-08
Firmicutes	Clostridia	Clostridiales	Ruminococcaceae	*Ruminococcaceae_UCG-013*	0,3%	2,21	3,2E-03
Firmicutes	Clostridia	Clostridiales	Ruminococcaceae	*Ruminococcaceae_UCG-014*	1,6%	4,16	7,7E-04
Firmicutes	Clostridia	Clostridiales	Ruminococcaceae	*Subdoligranulum*	2,3%	0,48	7,9E-04
Firmicutes	Erysipelotrichia	Erysipelotrichales	Erysipelotrichaceae	*Catenibacterium*	0,4%	3,31	3,3E-02
Firmicutes	Erysipelotrichia	Erysipelotrichales	Erysipelotrichaceae	*Erysipelotrichaceae_UCG-003*	0,4%	0,44	1,7E-02
Firmicutes	Negativicutes	Selenomonadales	Veillonellaceae	*Megasphaera*	0,6%	5,01	9,9E-03
Firmicutes	Negativicutes	Selenomonadales	Veillonellaceae	*Veillonella*	1,3%	0,22	3,0E-04
Tenericutes	-	-	-	-	0,3%	9,23	4,8E-11
Tenericutes	Mollicutes	-	-	-	0,3%	8,98	1,0E-09
Tenericutes	Mollicutes	Mollicutes_RF39	-	-	0,1%	7,89	1,1E-07

(1) Proportion is at the lowest listed taxonomic level. Proportions are from reads with known taxonomic assignation at the given level (i.e. therefore the *Bifidobacterium* genus has a mean proportion of 6.0% whereas its higher taxonomic levels make up for a smaller proportions - at the level of genus the total of taxonomically assigned reads is lower than at the higher levels).

(2) For the term of *Blastocystis* positivity versus negativity in a model adjusted for nationality.

Figure S1. Screening of eukaryotic parasites in stool using 18S rDNA profiling.

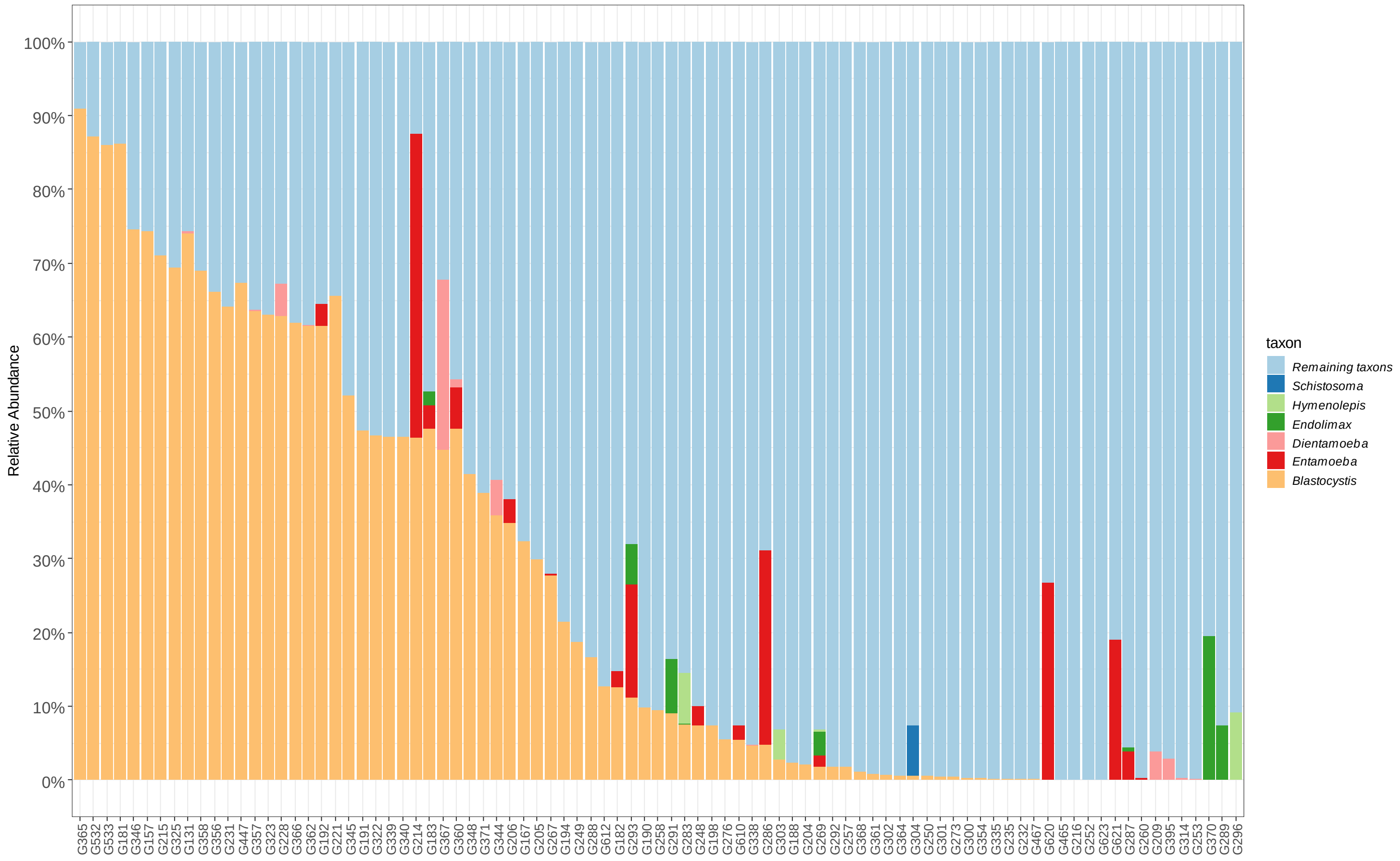

Only samples positive for at least one parasite are shown; samples are sorted in decreasing order by *Blastocystis* abundance. Signals from multicellular parasites presumably originate from ova. The signal under "remaining taxons" belongs to other eukaryotes (human cells, fungi, plant DNA from food etc.)

Figure S2. Genotyping reactions, with the flowchart of failed reactions

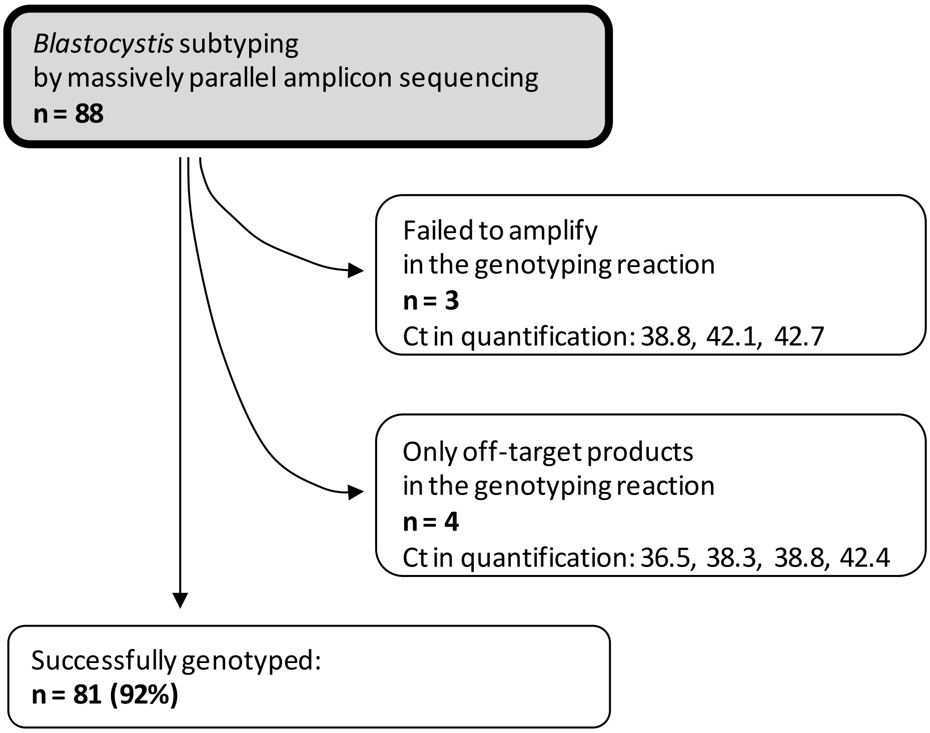

Figure S3. ***Blastocystis* subtypes and intra-subtype variations without reducing the number of ZOTUs**

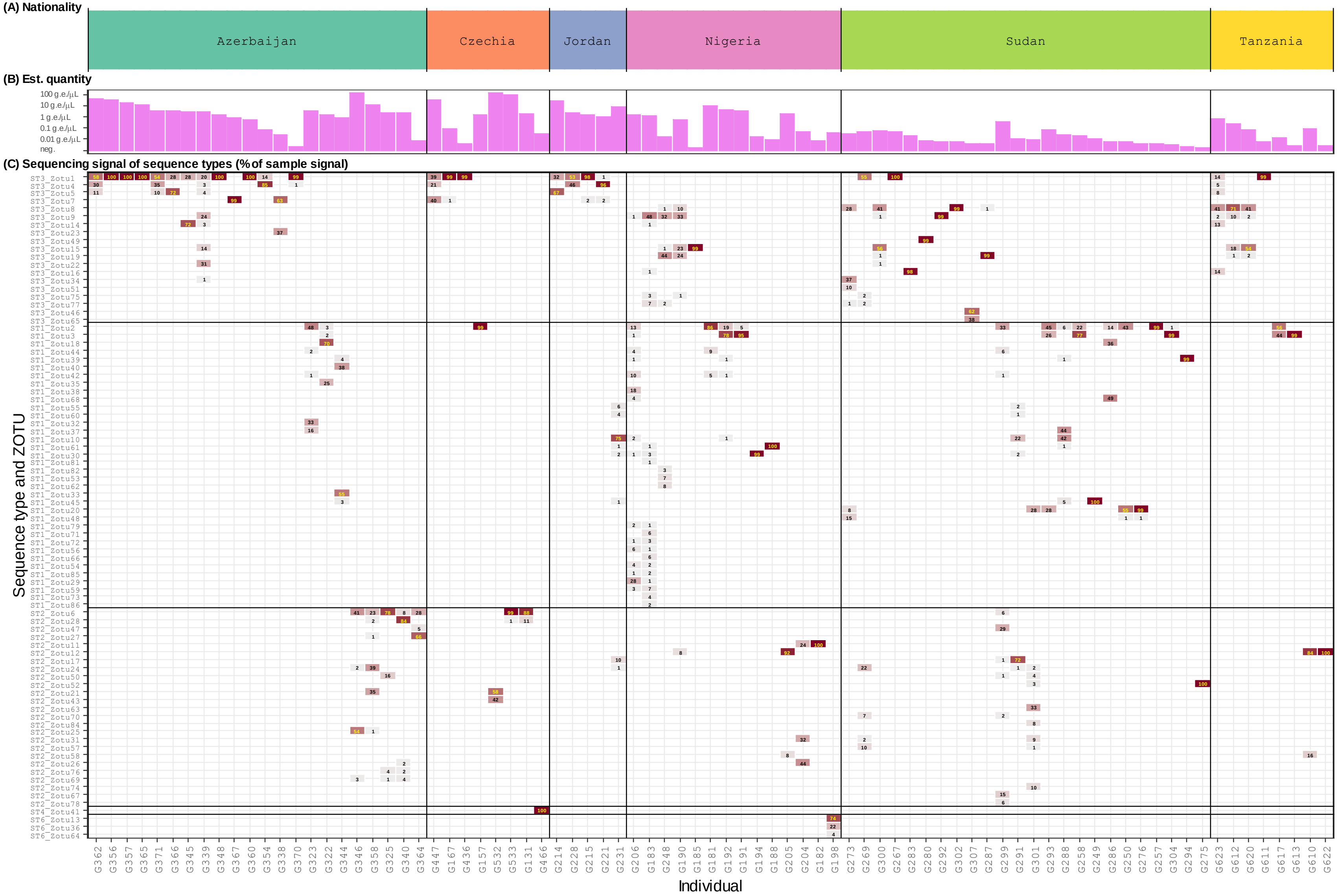

Figure S4. Phylogram of all ZOTUs in the study on the background of GenBank sequences representative of *Blastocystis* subtypes.

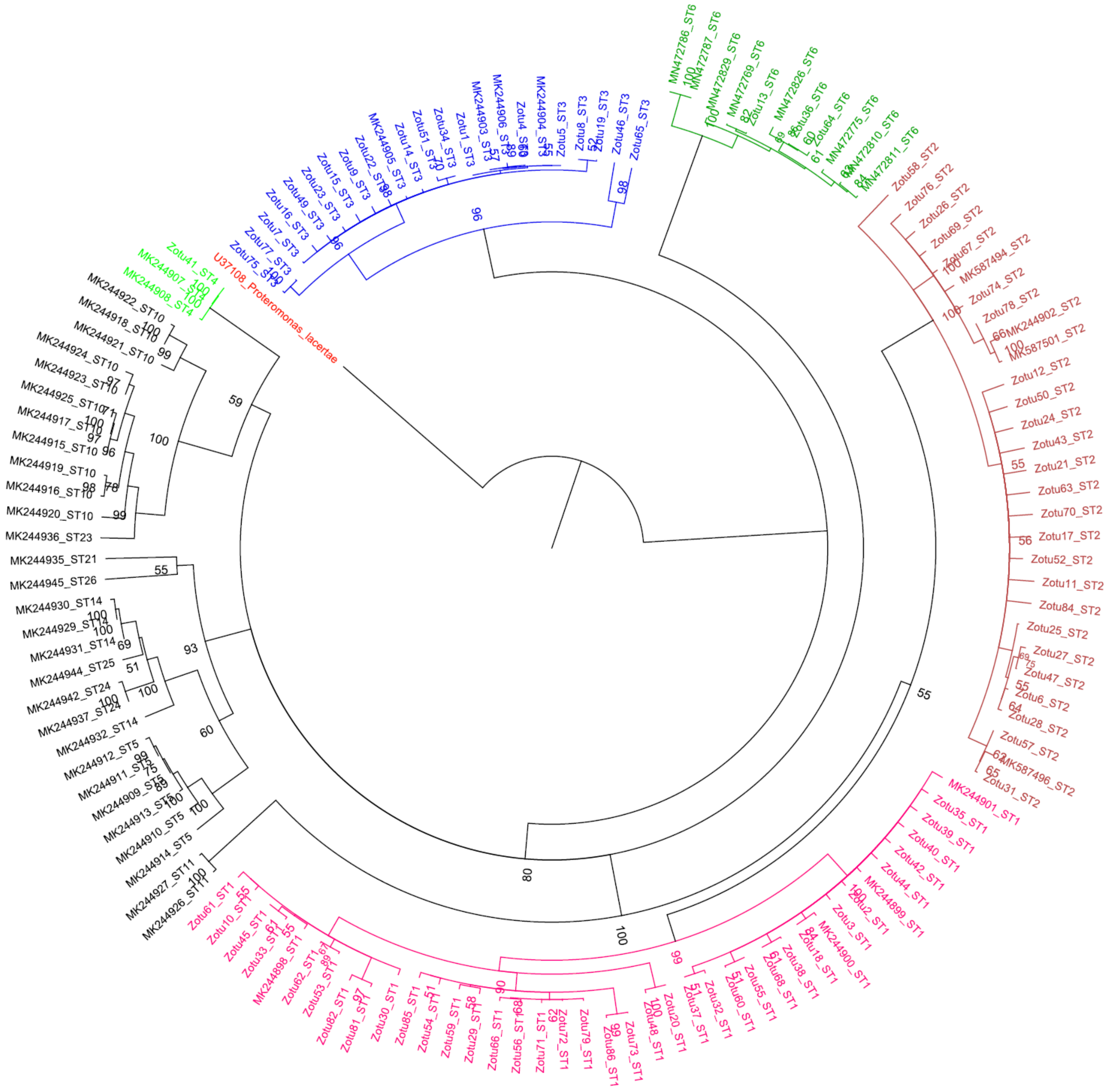

Figure S5. Alpha diversity of the bacteriome by positivity for *Blastocystis*

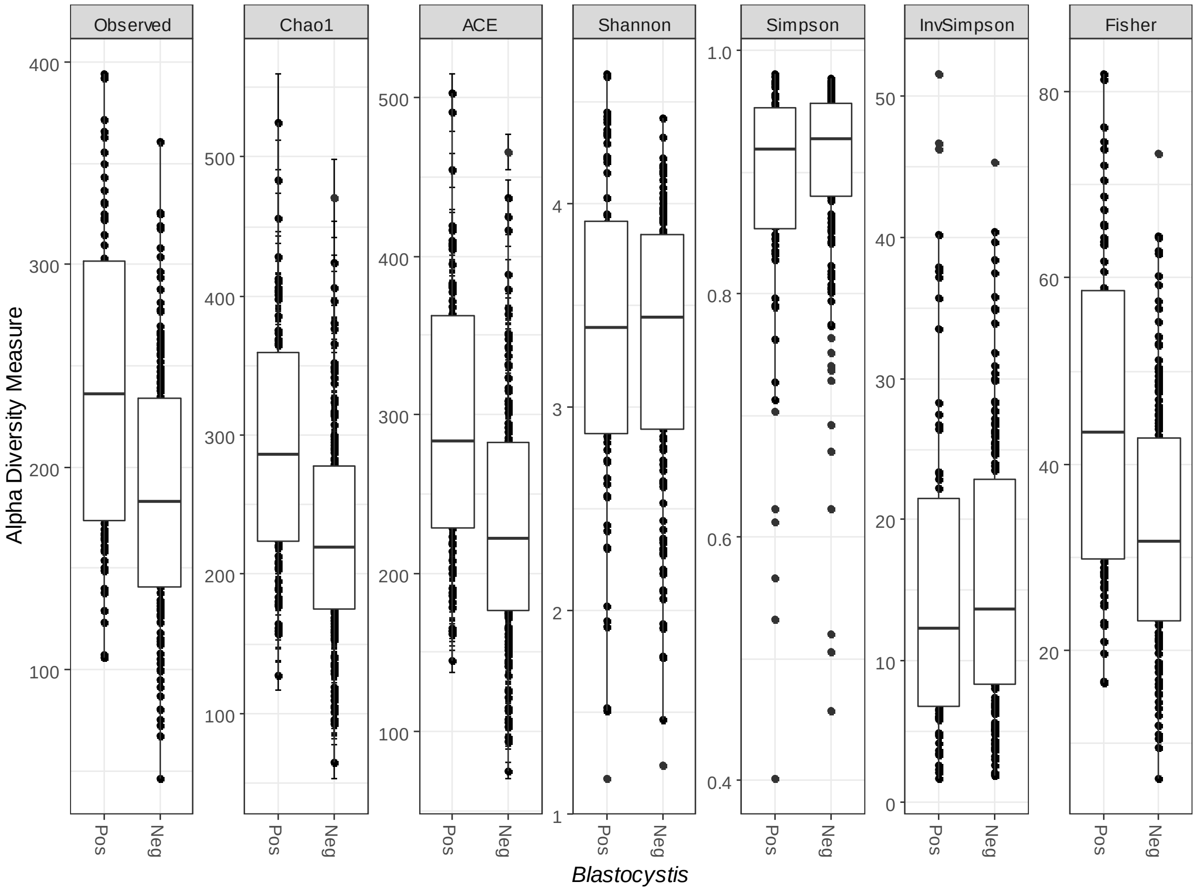

Figure S6. Heatmap showing the relative abundance of bacterial genera associated with *Blastocystis* positivity

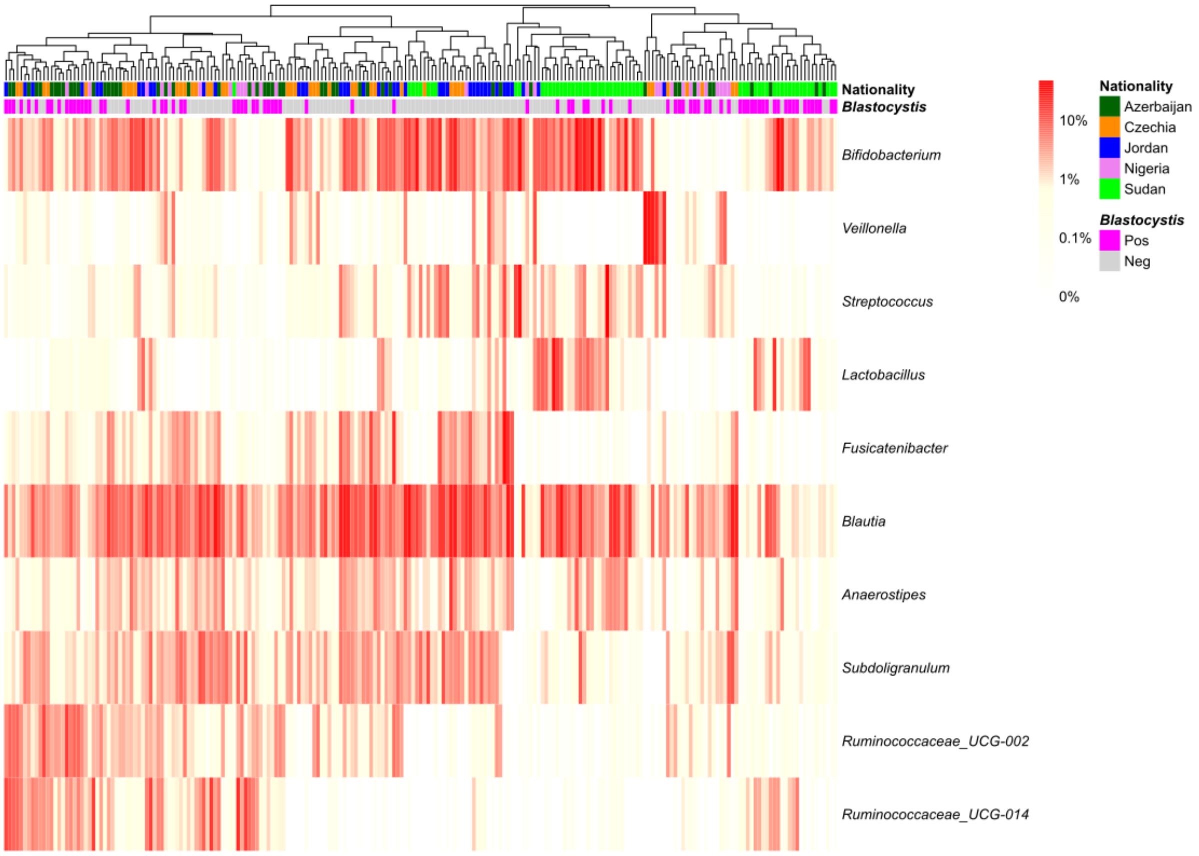

Only taxa significantly associated with *Blastocystis* are shown. The quantities are scaled to their maxima in order to be visible on a common color scale.

Figure S7. Abundance of bacterial genera associated with *Blastocystis* positivity.

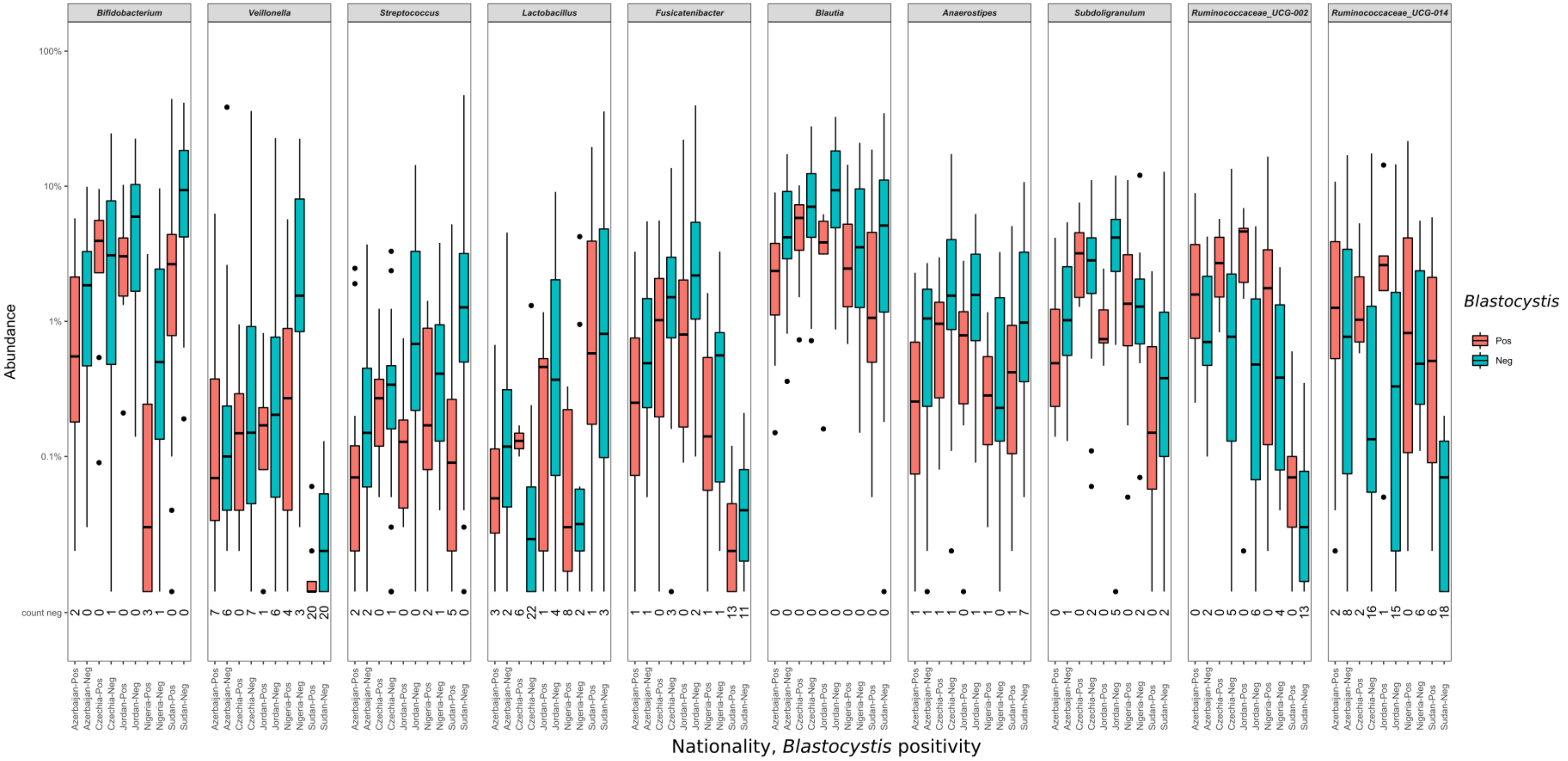

Each panel shows one genus, the horizontal axis is categorized by nationality and *Blastocystis* positivity. "Count neg" is the count of samples with zero abundance of the respective genus.

Figure S8. Comparison of specific real-time PCR for *Blastocystis* to the signals from the profiling of eukaryotic microorganisms.

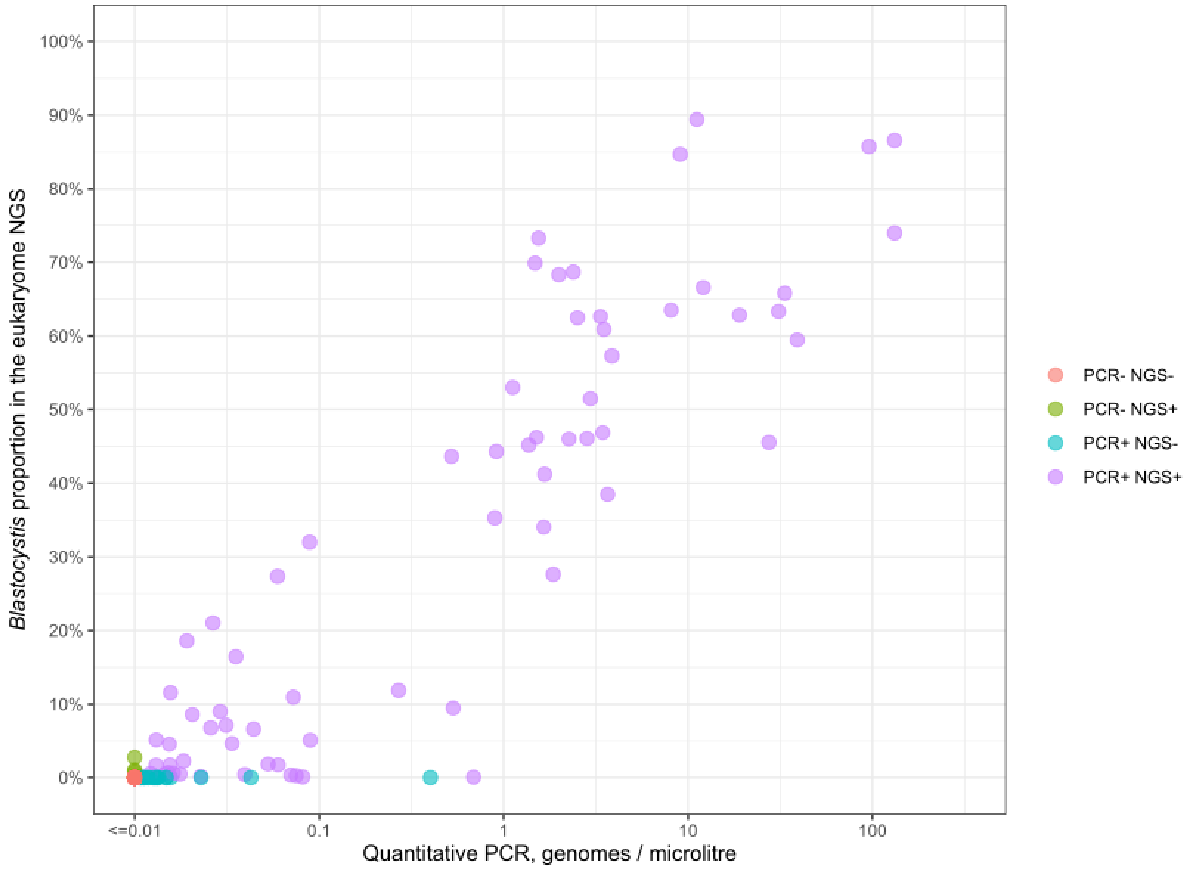

Supplementary Methods. Massively parallel 18S rDNA profiling of the eukaryotic parasitome

1. First round of amplification:

Primers. Underlined is the specific portion of the primer. Italics denotes the pad, bold the primary Illumina adaptor.

Designation	Sequence
H1183_1438_for5	**TCGTCGGCAGCGTC***AGATGTGTATAAGAGACAG*AATTTGACTCAACACGGG
H1183_1438_rev7	**GTCTCGTGGGCTCGG***AGATGTGTATAAGAGACAG*CATCACAGACCTGTTATTGC
H1422_1631_for5	**TCGTCGGCAGCGTC***AGATGTGTATAAGAGACAG*ATAACAGGTCTGTGATGC
H1422_1631_rev7	**GTCTCGTGGGCTCGG***AGATGTGTATAAGAGACAG*TACAAAGGGCAGGGACG
Euk18Sami_for5	**TCGTCGGCAGCGTC***AGATGTGTATAAGAGACAG*GTACACACCGCCCGTC
Euk18Sami_rev7	**GTCTCGTGGGCTCGG***AGATGTGTATAAGAGACAG*TGATCCTTCTGCAGGTTCACCTAC

Amplification and purification
Each sample was amplified in five independent reactions, containing the following combination of primers: (1) H1183_1438_for5 + H1183_1438_rev7; (2) H1422_1631_for5 + H1422_1631_rev7; (3) Euk18Sami_for5 + Euk18Sami_rev7; (4) H1183_1438_for5 + H1422_1631_rev7; (5) H1183_1438_for5 + Euk18Sami_rev7. Each PCR reaction contained in a total volume of 10 µl: 1x PCR buffer, 2.0 mM $MgCl_2$, 0.20 mM each dNTP, 450 nM each primer, 0.30 U Qiagen HotStar Taq polymerase, and 2 µl DNA template. The PCR program started with 15 minutes of denaturation at 95°C, and continued with 38 cycles of denaturation at 94°C, 30s, annealing at 55°C, 1 min, and synthesis 72°C, 30 s. The PCR was concluded with a final synthesis step of 5 minutes at 72°C.

The products of five reactions were pooled by sample, and purified with an equal volume of Ampure XT.

2. Second round of amplification (indexing), sequencing:
The indexing reactions contained 3µl of purified pooled products of the first PCR round, 1x KAPA HiFi HotStart Master Mix (Roche), 1.5 µl 7- and 5- indexing primer (combinatorial indexing primer sets *Nextera XT Index Kit v2 Set A and D,* FC-131-2001 and FC-131-2004, Illumina, that together create an indexing space of 384 combinations). Indexing was performed using a 8-cycle PCR program (denaturation 3 minutes at 95°C, then 8 cycles of 30 s at 95°C, 60 s at 55°C, 30 s at 72°C, concluding with final synthesis of 5 min at 72°C.

Clean-up and equalization followed. Sequencing was performed on a MiSeq machine with 2x250 reads kit version 2.

3. Bioinformatics
Reads were trimmed and filtered, and processed by the USEARCH61 pipeline with operational taxonomic units (OTUs) constructed at 97% similarity. Taxonomy was manually curated in 200 most abundant OTUs. The data were collated into a *phyloseq* object in R, and taxonomically aggregated at the level of genus. The signals were rarefied to 10,000 reads per sample, and OTUs below 0.05% abundance eliminated from each sample - this should decrease the effect of the known weak cross-bleed between indices due to index hopping. Further, extremely rare taxa were eliminated: we deleted OTUs that had neither over 5% in at least one sample nor 0.5% in 3 or more samples.
